# Identification of Reactive Metabolites of Acetaminophen and Saxagliptin in Human Hepatocytes and Hepatic Organoids

**DOI:** 10.3390/pharmaceutics18040483

**Published:** 2026-04-14

**Authors:** Im-Sook Song, Minyeong Pang, Min Seo Lee, Jihoon Lee, Kwang-Hyeon Liu, Min-Koo Choi, Han-Jin Park, Hyemin Kim, Hye Suk Lee

**Affiliations:** 1 College of Pharmacy and Research Institute of Pharmaceutical Sciences, Kyungpook National University, Daegu 41566, Republic of Korea; isssong@knu.ac.kr (I.-S.S.); legadema0905@knu.ac.kr (J.L.); dstlkh@knu.ac.kr (K.-H.L.); 2 BK21 FOUR Community-Based Intelligent Novel Drug Discovery Education Unit, Vessel-Organ Interaction Research Center (VOICE), Kyungpook National University, Daegu 41566, Republic of Korea; 3 College of Pharmacy, Dankook University, Cheon-an 31116, Republic of Korea; mignon@dankook.ac.kr (M.P.); minkoochoi@dankook.ac.kr (M.-K.C.); 4 Drug Metabolism and Bioanalysis Laboratory, College of Pharmacy, The Catholic University of Korea, Bucheon 14662, Republic of Korea; minseo.lee@catholic.ac.kr; 5 Department of Predictive Toxicology, Korea Institute of Toxicology, Daejeon 34114, Republic of Korea; hjpark@kitox.re.kr

**Keywords:** human pluripotent stem cell-derived hepatic organoids, plateable cryopreserved human hepatocytes, reactive metabolite, gluthathione conjugate, cysteine conjugate

## Abstract

**Objectives**: This study aims to identify the reactive metabolite of acetaminophen (AAP) and the cyanopyrrolidine metabolite of saxagliptin in human induced pluripotent stem cell-derived hepatic organoids (HHOs) and to compare them with human liver microsomes (HLMs) and plateable cryopreserved human hepatocytes (CHHs) to evaluate the feasibility of HHOs for reactive metabolite screening and metabolite profiling. **Methods**: AAP (50 μM) or sax-agliptin (50 μM) was incubated for 1 h at 37 °C in HLMs with or without NADPH-generating solution and 0.5 mM reduced glutathione (GSH). AAP (50 μM) was incubated for 24 h in HHOs and CHHs at 37 °C in a CO_2_ incubator. AAP and saxagliptin metabolites in the reaction mixtures were analyzed using ultra-performance liquid chromatography coupled with tandem mass spectrometry. **Results**: *N*-acetyl-*p*-benzoquinone imine (NAPQI) was identified in the incubation mixture of HLMs with AAP, and its levels were reduced in the presence of GSH, accompanied by increased formation of AAP–GSH adduct. Incubation of AAP with HHOs for 24 h resulted in the formation of NAPQI, AAP–GSH, AAP–glucuronide, and AAP–sulfate. Moreover, CYP1A2 induction using omeprazole treatment increased the formation of AAP and AAP–GSH conjugate from phenacetin, reflecting enhanced CYP1A2 activity in both CHHs and HHOs. The findings indicate that HHOs are a suitable platform for reactive metabolites, such as NAPQI and AAP–GSH adducts, under chronic exposure and metabolic modulator intervention. Additionally, CHHs and HHOs exhibited similar saxagliptin metabolite profiles after incubation with saxagliptin and generated cysteine conjugates of saxagliptin and its hydroxylated metabolite. **Conclusions**: HHOs system can be used as an in vitro model for screening reactive metabolites, comparable to those obtained with CHHs.

## 1. Introduction

Drugs have often been withdrawn following the emergence of severe hepatotoxicity signals, such as acute liver failure, during post-marketing surveillance—risks that are often undetected in clinical trials with limited sample sizes [1]. In addition to bromfenac, iproniazid, and troglitazone, drugs withdrawn from the market due to drug-induced liver injury (DILI) include nefazodone, ximelagatran, lumiracoxib, sitaxentan, trovafloxacin, alatrofloxacin, ibufenac, benoxaprofen, and alpidem [2,3]. Metabolic rate critically influences the duration of pharmacological activity and safety, as rapid increases in systemic drug exposure may elevate toxicity. Specifically, reactive metabolites generated during cytochrome P450 (CYP)-mediated drug metabolism are chemically unstable and electrophilic, enabling covalent binding to biological macromolecules, such as nucleic acids and proteins [3]. This process, termed covalent modification or adduct formation, represents a key mechanism underlying hepatotoxicity signals, including DILI [4].

The body employs protective mechanisms, such as enzymatic detoxification systems (e.g., glutathione [GSH] S-transferases), to neutralize reactive intermediates before significant damage occurs [5]. However, toxicity may occur when metabolite production exceeds detoxification capacity or in individuals with genetic variations in these protective enzymes. Additionally, DILI is a major cause of post-marketing drug withdrawals, as rare but severe hepatic adverse effects are often not detected in clinical trials, which typically enroll <3000 participants [6]. Therefore, early confirmation of reactive or toxic metabolites or their GSH or cysteine conjugates is critical in drug development.

Acetaminophen (AAP) overdose induces hepatotoxicity via its metabolite, *N*-acetyl-*p*-benzoquinone imine (NAPQI), a highly reactive compound that is normally detoxified by GSH. During overdose, GSH depletion allows NAPQI to covalently bind hepatic proteins, particularly in mitochondria, thereby disrupting energy production and inducing oxidative stress, which leads to hepatocellular necrosis and acute liver failure [7,8,9]. CYP2E1 and CYP1A2 mediate NAPQI bioactivation, with CYP1A2 contributing more substantially at high (toxic) AAP doses, particularly when CYP2E1 becomes saturated [10]. Studies using knockout mice show that animals lacking CYP1A2 and CYP2E1 are highly resistant to AAP-induced hepatotoxicity, confirming the essential roles of these enzymes [11]. Consequently, identifying the reactive metabolite NAPQI and its GSH conjugates is crucial for understanding hepatotoxicity mechanisms. Primary human hepatocytes, HepaRG cells, and human liver microsomes (HLMs) are commonly used to assess drug safety, evaluate hepatotoxic potential, and screen for reactive metabolites [12].

Human hepatic organoids (HHOs) are an advanced platform for recapitulating a physiologically relevant environment and maintain stable function for several weeks. HHOs derived from patients with DILI may help predict idiosyncratic reactions [13]. Hepatotoxicity screening of various biological molecules and known hepatotoxic drugs is often conducted in HHOs and compared with human hepatocytes [14,15]. However, the identification of reactive metabolites in the HHOs remains limited. Therefore, this study aims to identify NAPQI and its GSH conjugate in AAP-treated HHOs and compare them to HLMs and cryopreserved human hepatocytes (CHHs).

Saxagliptin, a potent, selective dipeptidyl peptidase-4 (DPP-4) inhibitor, is widely used to treat type 2 diabetes mellitus. Saxagliptin features a pyrrolidine ring with a cyano group, which forms a reversible covalent binding with the catalytic serine residue in the DPP-4 active site but dissociates slowly, allowing for sustained inhibition with once-daily dosing. Therefore, the recommended dose is 2.5–5.0 mg once daily [16]. While large trials report similar liver enzyme levels with saxagliptin and placebo, Thalha et al. report a rare case in Malaysia where a patient developed significant hepatotoxicity, characterized by steatohepatitis with cholestasis and elevated alanine aminotransferase and gamma-glutamyl transferase levels, after starting Kombiglyze (combination of saxagliptin 5 mg and metformin 1000 mg), with liver function normalizing after discontinuation. This case highlights the need for vigilance when using this drug combination, particularly given the greater hepatic metabolism of saxagliptin compared to the other DPP-4 inhibitors [17,18]. Saxagliptin forms reactive thiazoline-containing cysteine and GSH adducts (M3–M6) in rat liver microsomes and in vivo rats, covalently binding to liver proteins, suggesting a potential mechanism for DILI. This reactivity arises from its cyanopyrrolidine structure, similar to vildagliptin, raising concerns of hepatotoxicity, although clinical relevance remains unclear [18,19]. Therefore, this study also aims to identify the cyanopyrrolidine metabolite of saxagliptin in HHOs and compare it with HLMs and CHHs and to assess the feasibility of HHOs as a tool for reactive metabolite screening and metabolite profiling.

## 2. Materials and Methods

### 2.1. Materials

Ultrapooled HLMs and plateable CHHs from three donors (Lot No. 303, 52-year-old Caucasian female; Lot No. 321, 58-year-old Caucasian female; and Lot No. 337A, 58-year-old Caucasian male) were purchased from Corning Life Sciences (Woburn, MA, USA).

An NADPH-generating solution (1 mM NADPH, 5 mM glucose-6-phosphate, 1 mM NADP+, and 1 U glucose-6-phosphate dehydrogenase), Matrigel, fetal bovine serum (FBS), ultralow attachment 24- and 96-well plate, and Biocoat Collagen type I-coated 48- and 96-well plates were purchased from Corning Life Sciences (Woburn, MA, USA). Trizol, cryopreserved hepatocyte recovery and plating media were purchased from Invitrogen (Carlsbad, CA, USA). Advanced DMEM/F12, 100× penicillin/streptomycin, 100× GlutaMAX, 100× HEPES, 100× N2, and 50× B27 were purchased from Gibco Inc. (Billings, MT, USA). BMP7, FGF10, FGF19, and HGF were obtained from PeproTech (Cranbury, NJ, USA).

Bovine serum albumin (BSA), DAPT, dexamethasone, *N*-acetyl-L-cysteine, [Leu^15^]-gastrin I human, nicotinamide, A83–01, forskolin, CHIR99021, AAP, GSH, potassium phosphate dibasic trihydrate, potassium phosphate monobasic, phenacetin, MgCl_2_, NAPQI, omeprazole, William’s Medium E, and Accutase were obtained from Sigma-Aldrich Co. (St. Louis, MO, USA). Acetonitrile, methanol, and water of HPLC grade were purchased from Fischer Scientific (Fair Lawn, NJ, USA). TaqMan^®^ RNA-to-C_T_™ 1-Step Kit, TaqMan^®^ Gene Expression Assays, and gene-specific probes and primers for real-time reverse transcription polymerase chain reaction (RT-PCR) were obtained from Applied Biosystems (Foster City, CA, USA). All other reagents were of cell culture grade or analytical grade.

### 2.2. Generation of Human Hepatic Organoids

#### 2.2.1. Generation of Human Endoderm Organoids and HHO Differentiation into Human Hepatic Organoids

HHOs were derived from HDF01-human induced pluripotent stem cells (HDF hPSCs) [20] and H9 human embryonic stem cells (H9 hESCs) [21], respectively. The generation of human hepatic endodermal organoids (HEOs) and the differentiation into HHOs have been described previously (Supplementary Figure S1A) [22,23]. Cryopreserved HEOs were thawed and cultured at 2 × 10^4^ cells per well in a low-attachment 24-well plate using expansion medium (EM) comprising Advanced DMEM/F12 supplemented with 1× GlutaMAX, 10 mM HEPES, 1× N2, 1× B27, 0.1% BSA, 1.25 mM *N*-acetyl-L-cysteine, 10 nM [Leu15]-gastrin I human, 10 mM nicotinamide, 5 μM A83–01, 10 μM forskolin, 3 μM CHIR99021, 50 ng/mL FGF10, 25 ng/mL HGF, and 5% Matrigel. For HEO passaging, HEOs were recovered from Matrigel, dissociated into single cells using Accutase, and split at a 1:6 ratio every 6–7 days using EM.

HEOs were differentiated into HHOs for 21 days, including an initial expansion phase. Cells were seeded at 2 × 10^4^ cells per well in EM containing 25 ng/mL BMP7 for 5–7 days, followed by culture in differentiation medium (DM) for 15 days at 37 °C in 5% CO_2_. DM comprised Advanced DMEM/F12 supplemented with 1× GlutaMAX, 10 mM HEPES, 1× B27, 0.1% BSA, 1.25 mM *N*-acetyl-L-cysteine, 10 nM [Leu^15^]-gastrin I human, 0.5 μM A83–01, 10 μM DAPT, 3 μM dexamethasone, 100 ng/mL FGF19, 25 ng/mL BMP7, 25 ng/mL HGF, and 5% Matrigel. The medium was replaced every 3 days.

#### 2.2.2. Marker Expression of Human Hepatic Organoids

For reverse transcription-quantitative polymerase chain reaction (RT-qPCR), total RNA from HHOs was extracted using TRIzol and reverse-transcribed with GoScript™ Reverse Transcription Mix (Promega, Madison, WI, USA). Real-time PCR analysis was performed using SYBR Green Real-Time PCR Master Mix (Promega, Carlsbad, CA, USA) on a StepOnePlus Real-Time PCR System (Thermo Fisher Scientific, Waltham, MA, USA) as described previously [23]. Supplementary Table S1 shows primers used for RT-qPCR. For immunofluorescence, HHOs were fixed with 4% paraformaldehyde, permeabilized with 0.1% Triton X, and blocked with 5% normal serum (Jackson ImmunoResearch, West Grove, PA, USA). Primary antibodies against hepatocyte markers (i.e., monoclonal anti-HNF4A [1:200, Thermo Fisher Scientific, Waltham, MA, USA] and anti-CK8 [1:200, Abcam, Cambridge, MA, USA]) were incubated overnight at 4 °C, followed by incubation with fluorescent-conjugated secondary antibodies for 1 h at 25 °C. Nuclei were stained with 4′,6-diamidino-2-phenylindole (DAPI; Chem Cruz, Dallas, TX, USA). Fluorescence images were obtained with a Zeiss LSM 800 confocal microscope (Carl Zeiss, Oberkochen, Germany) at 20× magnification [14]. 

### 2.3. Identification of Reactive Metabolites of Acetaminophen in Human Liver Microsomes and Human Hepatic Organoids

#### 2.3.1. Incubation of Acetaminophen with Human Liver Microsomes

A 100 µL aliquot of 50 mM potassium phosphate buffer (pH 7.4) containing pooled HLMs (0.25 mg/mL), an NADPH-generating solution, 10 mM MgCl_2_, and 50 μM AAP was incubated for 1 h at 37 °C in the presence or absence of 0.5 mM GSH. The reaction was then quenched with 400 μL of ice-cold acetonitrile containing 1 ng/mL berberine as an internal standard, and the mixtures were centrifuged at 13,000 rpm for 5 min. A 5 μL aliquot of the supernatant was injected into an ultra-performance liquid chromatography coupled with tandem mass spectrometry (UPLC–MS/MS) system to monitor AAP metabolites.

#### 2.3.2. Incubation of Acetaminophen with Human Hepatic Organoids

After 21 days of differentiation, HHOs were suspended in 0.5 mL of organoid basal media (i.e., Advanced DMEM/F12 supplemented with 1% penicillin/streptomycin, 1% GlutaMAX, 10 mM HEPES, and 0.1% BSA) to eliminate the effects of growth factors and Matrigel. The organoid suspension was transferred to a 24-well ultralow attachment plate at a density of 2 × 10^4^ cells/well and was treated with 50 μM AAP for 24 h at 37 °C in 5% CO_2_ incubator. After 24 h, the reaction was quenched with 500 μL of ice-cold acetonitrile. An aliquot (100 μL) of the resulting supernatant was mixed with 200 μL of acetonitrile containing 1 ng/mL berberine, centrifuged at 13,000 rpm for 5 min, and a 5 μL aliquot of the final supernatant was injected into the LC–MS/MS system to monitor AAP metabolites. For cell viability measurement, a 100 μL aliquot of organoid suspension containing 50 μM AAP was transferred to a 96-well ultralow attachment plate at a density of 0.5 × 10^4^ cells/well and cultured for 24 h. Cell viability was measured using the CellTiter-Glo^®^ 3D Cell Viability Assay (Promega, Madison, WI, USA), according to the manufacturer’s instructions. Viability was expressed as the percentage of live cells compared with the control group.

#### 2.3.3. Identification of Acetaminophen Metabolites

AAP metabolites were identified using a TSQ Altis Plus Triple Quadrupole MS coupled to a Vanquish UPLC system (Thermo Fisher Scientific, Waltham, MA, USA). HLM samples were separated on an Atlantis^®^ HILIC Silica column (2.1 × 100 mm, 3 μm; Waters, Milford, MA, USA) using a gradient of deionized water containing 0.1% formic acid (A) and acetonitrile containing 0.1% formic acid (B) at a 0.4 mL/min flow rate. The gradient program was as follows: 0–2.0 min, 90% B; 2.0–2.5 min, 90–10% B; 2.5–5.0 min, 10% B; 5.0–5.5 min, 10–90% B; and 5.5–15.0 min, 90% B. Autosampler and column temperatures were set at 4 °C and 40 °C, respectively. Electrospray source settings were as follows: ion transfer tube temperature, 325 °C; vaporizer temperature, 200 °C; sheath gas flow, 50 Arb; auxiliary gas flow, 10 Arb; sweep gas flow, 1 Arb; and spray voltage, 3500 V. AAP and its metabolites [i.e., AAP, NAPQI, AAP–GSH, AAP–glucuronide (AAP–G), and AAP–sulfate (AAP–S)] were identified using full MS, product ion, and precursor ion scan modes under a positive-mode electrospray ionization (ESI) source via comparison with authentic standards (AAP and NAPQI) or previously published references (AAP–GSH, AAP–G, and AAP–S) [24,25,26].

Relative quantification of AAP metabolites in HLMs, HHOs, and CHHs was performed under the analytical conditions described below. Analytes were separated on a Luna^®^ Omega Polar C18 (2.1 × 100 mm, 3 μm; Phenomenex, Torrance, CA, USA) using an isocratic elution of 40% deionized water with 0.1% formic acid and 60% acetonitrile with 0.1% formic acid at a 0.15 mL/min flow rate, with a total run time of 5 min. Autosampler and column temperatures were set at 4 °C and 30 °C, respectively. Electrospray source settings were as follows: ion transfer tube temperature, 325 °C; vaporizer temperature, 200 °C; sheath gas flow, 50 Arb; auxiliary gas flow, 10 Arb; sweep gas flow, 1 Arb; spray voltage, 4000 V. Multiple reaction monitoring (MRM) transitions for AAP metabolites were monitored under a positive-mode ESI source with the following transitions and collision energies: AAP (*m*/*z* 152.0 → 110.0, 15 V), NAPQI (*m*/*z* 150.0 → 108.0, 10 V), AAP–GSH (*m*/*z* 457.1 → 328.2, 10 V), AAP–G (*m*/*z* 328.1 → 152.1, 10 V), AAP–S (*m*/*z* 232.3 → 152.1, 10 V), and berberine (*m*/*z* 336.1 → 320.0, 15 V).

### 2.4. Identification of Reactive Metabolites of Acetaminophen Following the Induction of CYP1A2 in Human Hepatocyte Organoids and Cryopreserved Human Hepatocytes

#### 2.4.1. Incubation of Phenacetin Followed by CYP1A2 Induction in Cryopreserved Human Hepatocytes

Plateable CHHs were thawed in cryopreserved hepatocyte recovery medium following the protocol of the manufacturer [27]. The thawed hepatocytes were then seeded onto collagen type I-coated 48-well plates at 2 × 10^4^ cells/well in 250 μL of hepatocyte plating medium containing FBS and incubated for 4 h at 37 °C in 5% CO_2_. The plating medium was replaced with hepatocyte culture medium containing FBS and 0.25 mg/mL Matrigel, and the cells were cultured for 24 h at 37 °C in 5% CO_2_. Subsequently, the hepatocytes were treated with omeprazole (0, 1, 5, 25, 50, or 100 μM), a representative AhR inducer, for 48 h at 37 °C in 5% CO_2_.

After the 48 h induction period, the effects of CYP1A2 mRNA expression and activity on AAP metabolism were evaluated using phenacetin, a probe substrate drug for CYP1A2. For mRNA analysis, CHHs were lysed with 500 µL of TRIzol reagent. Total RNA was extracted from cell lysates using a phenol–guanidine isothiocyanate method following the protocol of the manufacturer [27,28]. For phenacetin metabolite identification, 250 µL of William’s E medium containing 20 µM phenacetin was added to each well, followed by incubation for 4 h. The reaction was stopped with 250 μL of ice-cold acetonitrile containing 1 ng/mL berberine. A 100 μL aliquot of the resulting supernatant was mixed with 200 μL of acetonitrile and centrifuged at 13,000 rpm for 5 min. Finally, 5 μL of the supernatant was injected into the UPLC–MS/MS system to monitor phenacetin metabolites. For cell viability assessment, hepatocytes seeded onto collagen type I-coated 96-well plates at 0.5 × 10^4^ cells/well were treated with 100 μL aliquot of hepatocyte culture medium containing 0.25 mg/mL Matrigel and omeprazole (0, 1, 5, 25, 50, or 100 μM) for 48 h. Viability was measured using the CellTiter-Glo^®^ 3D Cell Viability Assay following the instructions of the manufacturer.

#### 2.4.2. Incubation of Phenacetin Followed by CYP1A2 Induction in Human Hepatic Organoids

After 21 days of differentiation, the HHOs were resuspended in 0.5 mL organoid basal medium containing omeprazole (0, 1, 5, 25, 50, or 100 μM) and seeded in an ultralow attachment 24-well plate at a density of 2 × 10^4^ cells/well and cultured for 48 h at 37 °C in a 5% CO_2_ incubator.

After the 48 h induction period, the effects of CYP1A2 mRNA expression and activity on AAP metabolism were evaluated. For mRNA analysis, HHOs were lysed with 500 µL TRIzol reagent, and total RNA was extracted from the cell lysates using a phenol–guanidine isothiocyanate method according to the protocol of the manufacturer [27,28]. For phenacetin metabolite identification, 250 µL of organoid basal medium containing 20 µM phenacetin was added to each HHO well, followed by incubation for 4 h. The reaction was stopped by adding 250 μL of ice-cold acetonitrile containing 1 ng/mL berberine as an internal standard. A 100 μL aliquot of the resulting supernatant was mixed with 200 μL of acetonitrile and centrifuged at 13,000 rpm for 5 min. Finally, 5 μL of the supernatant was injected into the UPLC–MS/MS system to monitor phenacetin metabolites. For cell viability assessment, a 100 μL aliquot of organoid suspension containing omeprazole (0, 1, 5, 25, 50, or 100 μM) was transferred into an ultralow attachment 96-well plate at a density of 0.5 × 10^4^ cells/well and cultured for 48 h. Viability was measured using the CellTiter-Glo^®^ 3D Cell Viability Assay according to the instructions of the manufacturer.

#### 2.4.3. RNA Purification and Reverse Transcription-Polymerase Chain Reaction Analysis

Total RNA concentration and purity of HHO and CHH samples were determined by measuring absorbance at 260/280 nm using a NanoVue Plus spectrophotometer (GE Healthcare Bio-Sciences Corp., Piscataway, NJ, USA). Samples were stored at −80 °C until analysis.

RT-qPCR was conducted using a Bio-Rad detection system (Hercules, CA, USA) with a TaqMan RNA-to-CT™ 1-Step Kit and TaqMan Gene Expression Assay (CYP1A2, Hs01070369_m1) according to the manufacturer’s protocol [27]. Total RNA (15 ng) per reaction was used under the following cycle conditions: 25 min reverse transcription at 48 °C, 15 min enzyme activation at 95 °C, 44 cycles of denaturation (15 s each) at 95 °C, and 1 min annealing/extension at 60 °C. Relative threshold cycle (ΔCt) values for all samples were normalized to the ΔCt of glyceraldehyde 3-phosphate dehydrogenase. Relative mRNA abundance was calculated for each sample using the comparative ΔΔCt method (2^−(ΔΔCt)^) [28,29].

#### 2.4.4. Metabolic Activity in Human Hepatic Organoids and Cryopreserved Human Hepatocytes

After 21 days of differentiation, the HHOs were suspended in 0.5 mL organoid basal medium containing CYP and UGT substrates (i.e., 20 μM phenacetin for CYP1A2, 1 μM midazolam for CYP3A4, 20 μM chlorozoxazone for CYP1A2, 2 μM SN-38 for UGT1A1, 2 μM *N*-acetylserotonin for UGT1A6, or 10 μM chrysin for SULT1A1), seeded into an ultralow attachment 24-well plate at a density of 2 × 10^4^ cells/well and cultured for 24 h at 37 °C in a 5% CO_2_ incubator.

Plateable CHHs were seeded in collagen type I-coated 48-well plates at 2 × 10^4^ cells/well in 250 μL of hepatocyte plating medium containing 0.25 mg/mL Matrigel, and the cells were cultured for 24 h at 37 °C in 5% CO_2_. After 24 h, the hepatocytes were treated with CYP and UGT substrates (i.e., 20 μM phenacetin for CYP1A2, 1 μM midazolam for CYP3A4, 20 μM chlorozoxazone for CYP1A2, 2 μM SN-38 for UGT1A1, 2 μM *N*-acetylserotonin for UGT1A6, or 10 μM chrysin for SULT1A1) for 24 h at 37 °C in a 5% CO_2_ incubator.

Reactions for CYP substrates in HHOs or CHHs were terminated by adding 500 μL ice-cold acetonitrile containing 1 ng/mL berberine and mixed vigorously for 10 min. The incubation mixtures were centrifuged at 13,000 rpm for 5 min at 4 °C, and 50 μL aliquots of each supernatant from CYP substrates were combined. After mixing, aliquots (5 μL) were analyzed using UPLC-MS/MS as described previously [30,31]. Analytes were separated on a Luna^®^ Omega Polar C18 (2.1 × 100 mm, 3 μm; Phenomenex, Torrance, CA, USA) using a gradient elution of 95% acetonitrile with 0.1% formic acid and 5% water with 0.1% formic acid at a flow rate of 0.15 mL/min. MRM transitions for metabolites were monitored with the following transitions, ion mode, and collision energies: AAP (*m*/*z* 152.0 → 110.0, positive, 15 V), 5-hydroxy midazolam (*m*/*z* 342.0 → 324.0, positive, 25 V), hydroxy chlorozoxazone (*m*/*z* 184.0 → 120.0, negative, 18 V), and berberine (*m*/*z* 336.1 → 320.0, positive, 15 V).

Similarly, the reactions for UGT substrates in HHO or CHHs were terminated by adding 500 μL ice-cold acetonitrile containing 1 ng/mL berberine and mixed vigorously for 10 min. The incubation mixtures were centrifuged at 13,000 rpm for 5 min at 4 °C, and 50 μL aliquots of each supernatant from UGT and SULT substrates were pooled. After mixing, aliquots (5 μL) were analyzed using UPLC-MS/MS as described previously [24,31,32]. MRM transitions for metabolites were monitored as follows: SN-38 glucuronide (*m*/*z* 569.0 → 393.0, positive, 30 V), *N*-acetylserotonin glucuronide (*m*/*z* 395.0 → 219.0, positive, 10 V), chrysin sulfate (*m*/*z* 335.3 → 255.0, positive, 20 V), and berberine (*m*/*z* 336.1 → 320.0, positive, 15 V).

### 2.5. Identification of Metabolites of Saxagliptin in Human Liver Microsomes, Human Hepatocyte Organoids, and Cryopreserved Human Hepatocytes

#### 2.5.1. Effect of Saxagliptin on the Viability of Human Hepatocyte Organoids and Cryopreserved Human Hepatocytes

For cell viability assessment, 100 μL aliquots of organoid suspension containing saxagliptin (0, 0.1, 1, 10, 50, and 500 μM) were transferred to an ultralow attachment 96-well plate at a density of 0.5 × 10^4^ cells/well and culture for 24 h.

Plateable CHHs were thawed and seeded in collagen type I-coated 96-well plates at 0.5 × 10^4^ cells/well. After 24 h, hepatocytes were treated with 100 μL hepatocyte culture medium containing saxagliptin (0, 0.1, 1, 10, 50, and 500 μM) for 24 h. Viability was measured using the CellTiter-Glo^®^ 3D Cell Viability Assay.

#### 2.5.2. Incubation of Saxagliptin with Human Liver Microsomes

A 100 µL aliquot of 50 mM potassium phosphate buffer (pH 7.4) containing pooled HLMs (0.25 mg/mL), an NADPH-generating solution, 10 mM MgCl_2_, and 50 μM saxagliptin was incubated at 37 °C for 1 h with or without 0.5 mM GSH. The reaction was stopped by adding 400 μL of ice-cold methanol containing 1 ng/mL berberine and the mixtures were centrifuged at 13,000 rpm for 5 min. The supernatants evaporated under an N_2_ gas stream. The residues were dissolved in 120 μL of 10% methanol, and an aliquot (3 μL) of each supernatant was injected into the UPLC-MS/MS system to monitor saxagliptin metabolites.

#### 2.5.3. Incubation of Saxagliptin with Human Hepatic Organoids

After 21 days of differentiation, HHOs were suspended in 0.5 mL of organoid basal media. The organoid suspension was transferred to a 24-well ultralow attachment plate at a density of 2 × 10^4^ cells/well and was treated with 50 μM saxagliptin for 24 h at 37 °C in a 5% CO_2_ incubator.

Subsequently, the reaction was terminated by adding 500 μL of ice-cold methanol containing 1 ng/mL berberine, followed by centrifugation at 13,000 rpm for 5 min. The supernatants were evaporated under an N_2_ gas stream, and the residues were reconstituted in 120 μL of 10% methanol. A 3 μL aliquot of each supernatant was injected into the UPLC–MS/MS system to monitor saxagliptin metabolites.

#### 2.5.4. Incubation of Saxagliptin with Cryopreserved Human Hepatocytes

Plateable CHHs were thawed in cryopreserved hepatocyte recovery medium following the protocol of the manufacturer. The thawed hepatocytes were seeded in collagen type I-precoated 48-well plates at 2 × 10^4^ cells/well in 250 μL hepatocyte plating medium and incubated for 4 h at 37 °C in 5% CO_2_ incubator. The plating medium was then replaced with hepatocyte plating medium containing 0.25 mg/mL Matrigel, and cells were cultured for 24 h at 37 °C in 5% CO_2_. Cells were subsequently incubated with 50 μM saxagliptin for 24 h at 37 °C in 5% CO_2_.

After 24 h, the reaction was stopped by adding 500 μL of ice-cold methanol containing 1 ng/mL berberine, followed by centrifugation at 13,000 rpm for 5 min. The supernatants were evaporated under an N_2_ gas stream, and the residues were reconstituted in 120 μL of 10% methanol. A 3 μL aliquot of each solution was injected into the UPLC–MS/MS system to monitor saxagliptin metabolites.

#### 2.5.5. Identification of Saxagliptin Metabolites

We applied the saxagliptin metabolite profiling methods and MS/MS fragmentation patterns established in our previous study [18] to guide saxagliptin metabolite analysis and interpret the product ion spectra in the present study. Saxagliptin metabolites were identified using a TSQ Altis Plus triple-quadrupole MS coupled to a Vanquish UPLC system (Thermo Fisher Scientific, Waltham, MA, USA). Chromatographic separation was conducted on a Kinetex^®^ C18 column (2.1 × 100 mm, 3 μm; Phenomenex, Torrance, CA, USA) using gradient elution with deionized water containing 0.1% formic acid (mobile phase A) and methanol with 10 mM ammonium formate (mobile phase B) at a flow rate of 0.2 mL/min. The gradient program was as follows: 0–2.0 min, 5% B; 2.0–4.0 min, 5–10% B; 4.0–9.0 min, 10–90% B; 9.0–9.1 min, 90–5% B; 9.1–17.0 min, 5% B. The autosampler and column temperatures were set to 4 °C and 30 °C, respectively. Electrospray source parameters were as follows: ion transfer tube temperature, 325 °C; vaporizer temperature, 200 °C; sheath gas flow, 50 Arb; auxiliary gas flow, 10 Arb; sweep gas flow, 1 Arb; and spray voltage, 4000 V. MRM transitions were acquired under positive ESI with the following precursor → product ion transitions and collision energies: saxagliptin and M1 (*m*/*z* 316.1 → 180.1, 25 V; distinguished by different retention times), M2 (*m*/*z* 332.1 → 196.0, 30 V), M3 (*m*/*z* 420.0 → 180.0, 30 V), M4 (*m*/*z* 436.1 → 436.1, 10 V), M5 (*m*/*z* 477.2 → 358.2, 10 V), M6 (*m*/*z* 493.2 → 374.2, 10 V), M7 (*m*/*z* 396.2 → 260.0, 10 V), and M8 (*m*/*z* 492.2 → 316.0, 10 V).

### 2.6. Data Analysis

E_max_ (maximum fold induction) and EC_50_ (concentration producing 50% of E_max_) were derived from dose–response curves for CYP1A2 mRNA induction [33], which were fitted to a sigmoidal model using SigmaPlot (version 10.0; Systat Software Inc., San Jose, CA, USA).

Data are presented as mean ± standard deviation (SD). Statistical analyses were performed using the nonparametric Mann–Whitney U test for two-group comparisons and Kruskal–Wallis test for comparisons among more than three groups in SPSS for Windows (version 26.0; IBM Corp., Armonk, NY, USA)

## 3. Results

### 3.1. Identification of Reactive Metabolites of Acetaminophen in Human Hepatic Organoids

Before assessing reactive metabolite formation, the hepatic phenotype of HHOs was confirmed. Organoids expressed key hepatic markers at both mRNA and protein levels, including ALB, AAT, HNF4A, TDO2, MDR1, MRP2, and CK8, demonstrating successful differentiation and functional hepatic characteristics (Supplementary Figure S1B,C).

**Figure 1:**
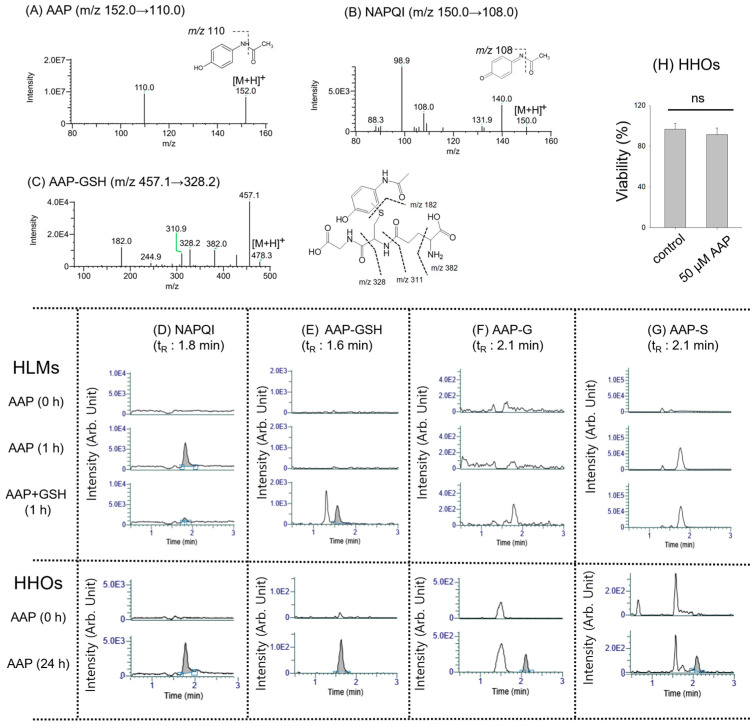
Product ion scan spectra of (**A**) AAP, (**B**) NAPQI, and (**C**) AAP–GSH obtained from authentic standards and from HLM samples. Representative MRM chromatograms of AAP metabolites such as (**D**) NAPQI, (**E**) AAP–GSH, (**F**) AAP–G, and (**G**) AAP–S, detected in HLMs under control conditions (0 h) and after 1 h incubation with AAP or AAP + GSH or in HHOs under control conditions (0 h) and after 24 h exposure to 50 μM AAP. (**H**) Viability of HHOs following the 24 h treatment with 50 μM AAP. Data are presented as mean ± SD (*n* = 3). Abbreviations: AAP, acetaminophen; NAPQI, *N*-acetyl-*p*-benzoquinone imine; GSH, glutathione; AAP–GSH, acetaminophen–glutathione conjugate; AAP–G, acetaminophen–glucuronide; AAP–S, acetaminophen–sulfate; HLMs, human liver microsomes; HHOs, human hepatic organoids; MRM, multiple reaction monitoring; ns, not significant compared with control group.

To evaluate the feasibility of HHOs as a screening platform for reactive metabolites, NAPQI and the AAP–GSH adduct were identified (Figure 1). These reactive AAP metabolites were first characterized in HLMs and then compared with HHOs. NAPQI (t_R_ of 1.8 min) was detected in HLM incubations with AAP using authentic AAP and NAPQI standards (Figure 1A,B,D).

Following AAP incubation with HLMs in the presence of NADPH and GSH, AAP–GSH conjugate was detected at *m*/*z* 457.0 → 328.1 with a t_R_ of 1.6 min (Figure 1C,E). The corresponding product ions of AAP–GSH were observed at *m*/*z* 457.1, 382.0, 328.2, 310.9, and 182.0 (Figure 1C). Since cofactors such as uridine 5′-diphospho-glucuronic acid (UDP-GA) and 3′-phosphoadenosine-5′-phosphosulfate (PAPS) were absent, AAP–G and AAP–S were not formed in HLMs (Figure 1F,G). Overall, the reactive AAP metabolites, NAPQI and AAP–GSH adduct, were detected in HLMs, consistent with previous reports [25,26].

Incubation of AAP with HHOs for 24 h resulted in the formation of NAPQI, AAP–GSH, AAP–G, and AAP–S (Figure 1D–G) without significant changes in cell viability (Figure 1H). Since the IC_50_ of AAP in human hepatocytes was ~10 mM [23,34], AAP treatment at a sublethal concentration (50 μM) for 24 h seemed to be appropriate for metabolite phenotyping and reactive metabolite identification. Collectively, the study results support the feasibility of HHO as a screening platform for identifying reactive metabolites such as NAPQI and the AAP–GSH adduct.

### 3.2. Effect of CYP1A2 Induction on the AAP Metabolites in Human Hepatic Organoids

**Figure 2:**
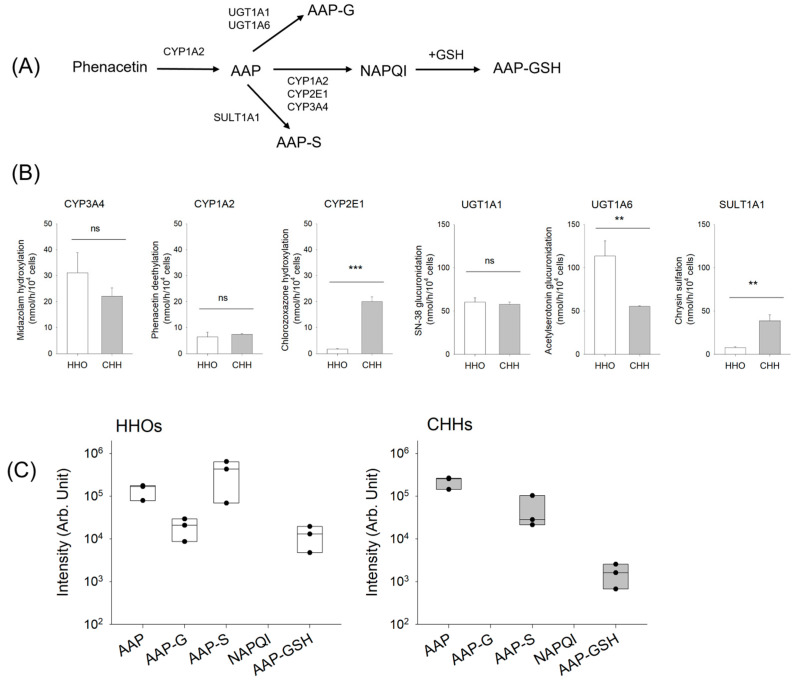
(**A**) Metabolic pathway of phenacetin. (**B**) Basal metabolic activities of CYP3A4, CYP1A2, CYP2E1, UGT1A1, UGT1A6, and SULT1A1 in HHOs (HDF HHO-P12) and CHHs (Lot 303). Data are presented as mean ± SD (*n* = 3). (**C**) Phenacetin metabolites detected in HHOs from three independent batches—HDF HHO-P14, HDF HHO-P38, and H9 HHO-P23—and in CHHs from three different donors (Lots 303, 321, and 337A). Data represent the mean of triplicate measurements for each organoid batch or individual donor. Abbreviations: AAP, acetaminophen; NAPQI, *N*-acetyl-*p*-benzoquinone imine; GSH, glutathione; AAP–GSH, acetaminophen–glutathione conjugate; AAP–G, acetaminophen–glucuronide; AAP–S, acetaminophen–sulfate; CYP, cytochrome P450; UGT, UDP-glucuronosyltransferase; HHOs, human hepatic organoids; CHHs, cryopreserved human hepatocytes; ** *p* < 0.01, *** *p* < 0.001; ns, not significant compared with HHO group.

Phenacetin was metabolized to AAP via CYP1A2, which subsequently underwent extensive phase II metabolism via glucuronidation and sulfation (Figure 2A). Approximately 5% of AAP was converted to NAPQI and then to AAP–GSH conjugate [35,36,37]. However, at high AAP concentrations, reactive metabolites NAPQI and AAP–GSH are produced in larger amounts, potentially causing liver injury [35,36,37]. Therefore, we compared AAP metabolism in HHOs with that in CHHs following CYP1A2 induction, the primary enzyme responsible for the phenacetin → AAP → NAPQI pathway.

Before evaluating AAP metabolism under CYP1A2 induction, we compared the basal activities of key AAP metabolizing enzymes such as CYP3A4, CYP1A2, CYP2E1, UGT1A1, UGT1A6, and SULT1A1 [38]. HHOs and CHHs exhibited metabolic activity for all enzymes investigated (Figure 2B). CYP3A4, CYP1A2, and UGT1A1 activities were comparable between HHOs and CHHs. HHOs exhibited higher CYP2E1 and UGT1A6 activities than CHHs, whereas CHHs showed higher SULT1A1 activity than HHOs (Figure 2B).

Following 24 h of phenacetin treatment in HHOs, phenacetin was metabolized to AAP, which was subsequently metabolized to AAP–G, AAP–S, and AAP–GSH. However, the reactive intermediate NAPQI was not detected in HHOs from three independent batches—HDF HHO-P14, HDF HHO-P38, and H9 HHO-P23—likely because it was rapidly conjugated with GSH to form AAP–GSH (Figure 2C). Compared to HHOs, incubation of phenacetin with CHHs from three different donors—Lots 303, 321, and 337A—resulted in the formation of AAP, AAP–S, and AAP–GSH (Figure 2C).

**Figure 3:**
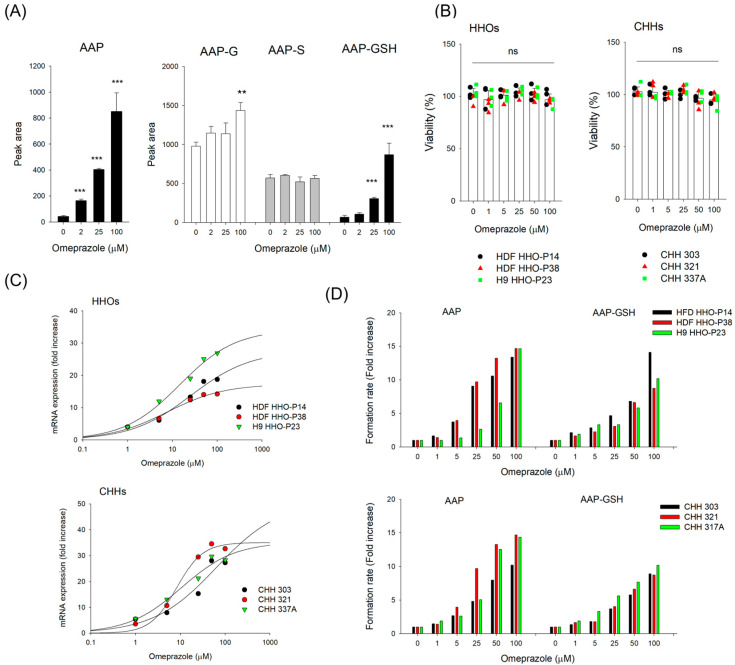
(**A**) Alterations in phenacetin metabolites in HHOs (HDF HHO-P12) following CYP1A2 induction by omeprazole over the concentration range of 0–100 μM. Data are presented as mean ± SD (*n* = 3). (**B**) Viability of HHOs and CHHs following 48 h of omeprazole treatment (0–100 μM). Data are presented as mean ± SD (*n* = 3) with triplicate measurements for each organoid batch or individual donor. Effects of prototypical inducer omeprazole on (**C**) CYP1A2 mRNA expression and (**D**) AAP and AAP–GSH formations after incubation after 4 h incubation with 20 μM phenacetin following 48 h of omeprazole treatment in HHOs from three independent batches—HDF HHO-P14, HDF HHO-P38, and H9 HHO-P23—and in CHHs from three different donors (Lots 303, 321, and 337A). Data represent the mean of triplicate measurements for each organoid batch or individual donor. Abbreviations: AAP, acetaminophen; AAP–GSH, acetaminophen–glutathione conjugate; CHHs, cryopreserved human hepatocytes; HHOs, human hepatic organoids. ** *p* < 0.01, *** *p* < 0.001 compared with vehicle treatment; ns, not significant among different concentration groups.

We investigated the effect of CYP1A2 induction on the formation of AAP–GSH—a reactive metabolite of phenacetin—in HHOs. After 48 h incubation with omeprazole (0–100 μM), AAP formation from phenacetin increased up to 19.7-fold in a concentration-dependent manner, while AAP–GSH formation rose 13.2-fold compared with the control group. However, omeprazole treatment did not significantly affect AAP–S formations. AAP-G increased 1.5-fold at the highest omeprazole concentration (100 μM) but showed no significant change at lower concentrations (Figure 3A). Collectively, CYP1A2 induction shifted phenacetin metabolism toward increased AAP formation and its reactive metabolite AAP–GSH, while AAP–G and AAP–S levels remained largely unchanged. The findings suggest that CYP1A2 induction may increase the risk of liver injury, even without changes in phenacetin concentration.

We then compared CYP1A2 inducibility after 48 h of omeprazole treatment in HHOs from three independent batches—HDF HHO-P14, HDF HHO-P38, and H9 HHO-P23—and in CHHs from three different donors (Lots 303, 321, and 337A). Omeprazole treatment (0–100 μM) for 48 h did not significantly affect the viability of HHOs or CHHs (Figure 3B). Omeprazole increased CYP1A2 mRNA levels in a concentration-dependent manner in HHOs and CHHs (Figure 3C). Table 1 presents induction parameters such as E_max_ and EC_50_, calculated from CYP1A2 dose–response curves. While E_max_ and EC_50_ values were similar between HHOs and CHHs, induction activity—calculated via E_max_/EC_50_—was approximately 2-fold higher in CHHs than in HHOs. Since CHHs are the gold standard for assessing the induction potential of drug-metabolizing enzymes, our findings demonstrate that HHOs provide a feasible platform for evaluating CYP1A2 induction and the risk of reactive metabolite formation.

Among phenacetin metabolites, the formation of AAP and AAP–GSH was involved in CYP1A2 metabolic activity (Figure 3A). Thus, we measured AAP and AAP–GSH formation in HHOs and CHHs under the same conditions as the CYP1A2 mRNA assessment. Consistent with the CYP1A2 mRNA increase, omeprazole treatment enhanced the formation rates of AAP and AAP–GSH in a concentration-dependent manner, with comparable levels observed between HHOs and CHHs (Figure 3D). These findings also suggest that omeprazole-induced CYP1A2 mRNA upregulation in HHOs and CHHs enhances AAP and AAP–GSH formation, potentially increasing the risk of liver injury. Collectively, HHOs represent a viable in vitro platform for identifying the reactive metabolites and their GSH conjugates, comparable to CHHs.

### 3.3. Comparison of Saxagliptin Metabolism in Human Hepatic Organoids and Human Hepatocytes

We previously identified saxagliptin–cysteine adducts in rat liver microsomes and in vitro in rats, and saxagliptin–GSH conjugate in rats, as reactive metabolites with potential to contribute to DILI [18]. Since these reactive metabolites can be relevant to the human system, we investigated their formation in HLMs, HHOs, and CHHs.

**Figure 4:**
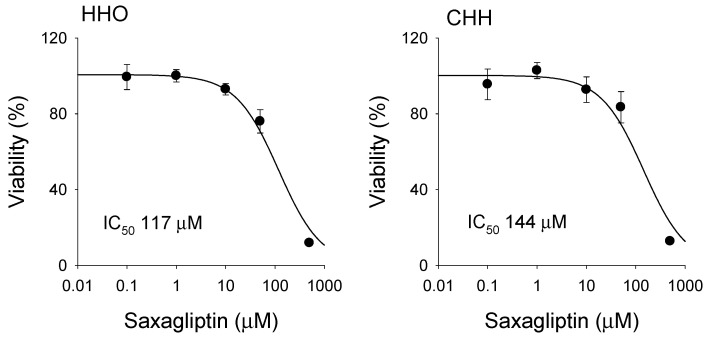
Viability of HHOs (HDF HHO-P12) and CHHs (Lot 303) following 24 h treatment of saxagliptin (0–100 μM). Data are presented as mean ± SD (*n* = 3). IC_50_ values of saxagliptin were calculated from the cell viability results. Abbreviations: HHOs, human hepatic organoids; CHHs, cryopreserved human hepatocytes; IC_50_, half-maximal inhibitory concentration.

Saxagliptin reduced the viability of HHOs and CHHs in a concentration-dependent manner following the 24 h treatment of saxagliptin (0–500 μM), yielding IC_50_ values of 117 μM and 144 μM, respectively (Figure 4). The results suggested that saxagliptin may induce hepatotoxicity at high concentrations. After treatment with 50 μM saxagliptin, the viabilities of HHOs and CHHs were 76.0 ± 6.2% and 83.5 ± 8.3%, respectively. Thus, the treatment of 50 μM saxagliptin for 24 h was selected as a sublethal concentration in both systems to investigate reactive metabolite formation in HHOs and CHHs.

**Figure 5:**
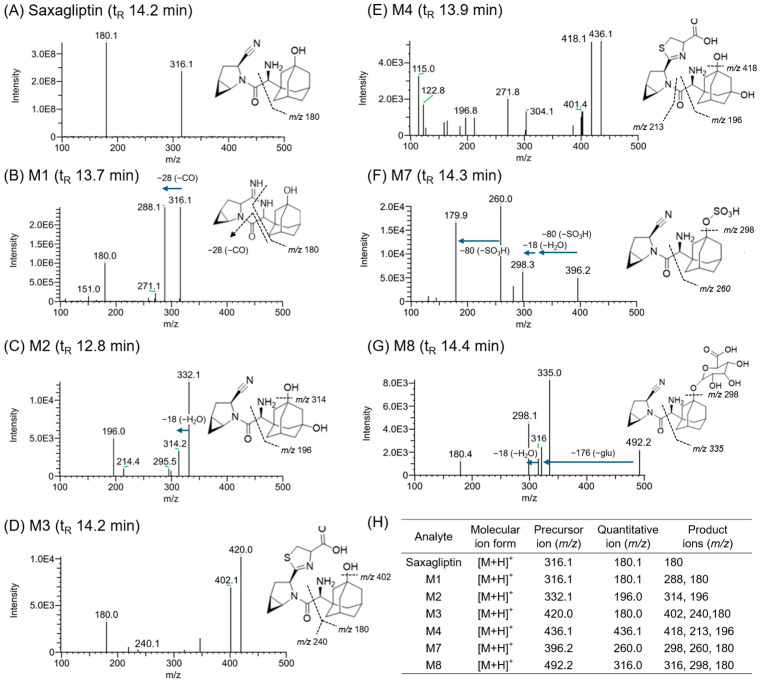
MS/MS spectra and fragmentation of (**A**) saxagliptin and its metabolites, (**B**) M1, (**C**) M2, (**D**) M3, (**E**) M4, (**F**) M7, and (**G**) M8. (**H**) Precursor ion ([M+H]^+^) and product ions of saxagliptin and its six metabolites after incubation with HLMs, HHOs (HDF HHO-P12), and CHHs (Lot 303). Abbreviations: HLMs, human liver microsomes; HHOs, human hepatic organoids; CHHs, cryopreserved human hepatocytes.

We identified six saxagliptin metabolites in hepatocyte samples after 24 h of incubation with 50 μM saxagliptin. Figure 5 shows the product ion scans of saxagliptin and its six metabolites. Saxagliptin exhibited product ions at *m*/*z* 316.1 ([M+H]^+^ ion) and *m*/*z* 180.1 (Figure 5A). The cyclic amide degradant M1 generated product ions at *m*/*z* 316.1, 288.1, and 180.1 (Figure 5B), consistent with previously reported fragmentation patterns [18,39]. Furthermore, 5-hydroxysaxagliptin produced a product ion at *m*/*z* 332.2, which was 16 amu higher than the [M+H]^+^ ion (*m*/*z* 316.1), along with fragment ions at *m*/*z* 314.2 and *m*/*z* 196.0 (Figure 5C), thereby confirming its identity. The cysteine conjugate of saxagliptin (M3) generated product ions at *m*/*z* 420.0, *m*/*z* 402.1, *m*/*z* 240.1, and *m*/*z* 180.0 (Figure 5D), consistent with previously reported data [18]. The cysteine conjugate of 5-hydroxysaxagliptin (M4) yielded product ions at *m*/*z* 436.1, *m*/*z* 418.1, *m*/*z* 213.1, and *m*/*z* 196.1 (Figure 5E), which matched previously published fragmentation patterns [18]. GSH conjugates of saxagliptin (M5) and 5-hydroxysaxagliptin (M6) were not detected in both HHOs and CHHs. Metabolites M7 and M8, representing the sulfate and glucuronide conjugates of saxagliptin, were detected in both HHOs and CHH samples. M7 yielded product ions at *m*/*z* 396.2 (80 amu higher than that of saxagliptin), *m*/*z* 298.3 (18 amu lower than that of saxagliptin, corresponding to loss of H_2_O), and *m*/*z* 179.9 (Figure 5F), thereby confirming its identity. M8 generated product ions at *m*/*z* 492.2 (176 amu higher than that of saxagliptin), *m*/*z* 316.0, *m*/*z* 298.1 (18 amu lower than that of saxagliptin, corresponding to loss of H_2_O), and *m*/*z* 180.4 (Figure 5G). Figure 5H shows the precursor, quantitative, and product ions of saxagliptin metabolites.

**Figure 6:**
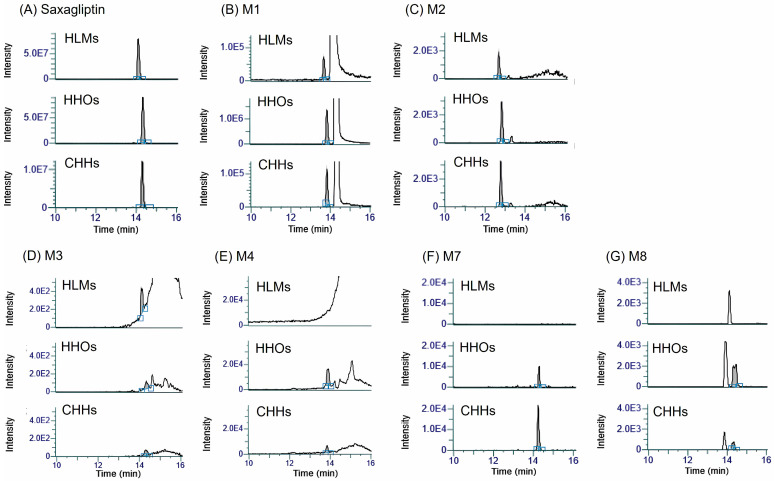
Extracted ion chromatograms of (**A**) saxagliptin and its metabolites, (**B**) M1, (**C**) M2, (**D**) M3, (**E**) M4, (**F**) M7, and (**G**) M8 in HLMs after 1 h incubation with saxagliptin and in HHOs (HDF HHO-P12), and CHHs (Lot 303) after 24 h incubation with saxagliptin. Chromatograms for the control samples (0 h), including those of M5 and M6, are provided in the Supplementary Materials. Abbreviations: HLMs, human liver microsomes; HHOs, human hepatic organoids; CHHs, cryopreserved human hepatocytes.

Figure 6 shows the MRM chromatograms obtained from the precursor and quantitative ions of saxagliptin metabolites in HLMs, HHOs, and CHHs after 1 h (HLMs) or 24 h incubation (HHOs and CHHs) with 50 μM saxagliptin. Following the incubation of saxagliptin, the remained saxagliptin peak observed in CHHs was the lowest among the three groups (Figure 6A), indicating greater metabolic activity in CHHs than in HLMs or HHOs. Subsequent metabolite identification was performed by comparing the MRM chromatograms of saxagliptin-incubated samples with the control samples (without saxagliptin) (Figure 6 and Figure S2). Metabolites M1, M2, and M3 were detected using the transitions *m*/*z* 316.1 → 180.1 and *m*/*z* 332.1 → 196.0, with retention times (t_R_) of 14.2, 13.7, and 12.8 min, respectively. Compared to HLMs, the relative abundances of M1 and M2 were higher in HHOs and CHHs, whereas that of M3 was lower in HHOs and CHHs (Figure 6B–D). The cysteine conjugate of 5-hydroxysaxagliptin (M4) was not formed, likely owing to the limited availability of cysteine in HLMs. GSH adduct of saxagliptin or M1 was not detected despite incubation of saxagliptin with HLMs in the presence of NADPH and GSH (Figure 6E). The sulfate and glucuronide conjugates of saxagliptin (M7 and M8) were also not found in HLMs owing to the absence of required cofactors such as UDP-GA, PAPS, or the cytosolic fraction (Figure 6F,G).

**Figure 7:**
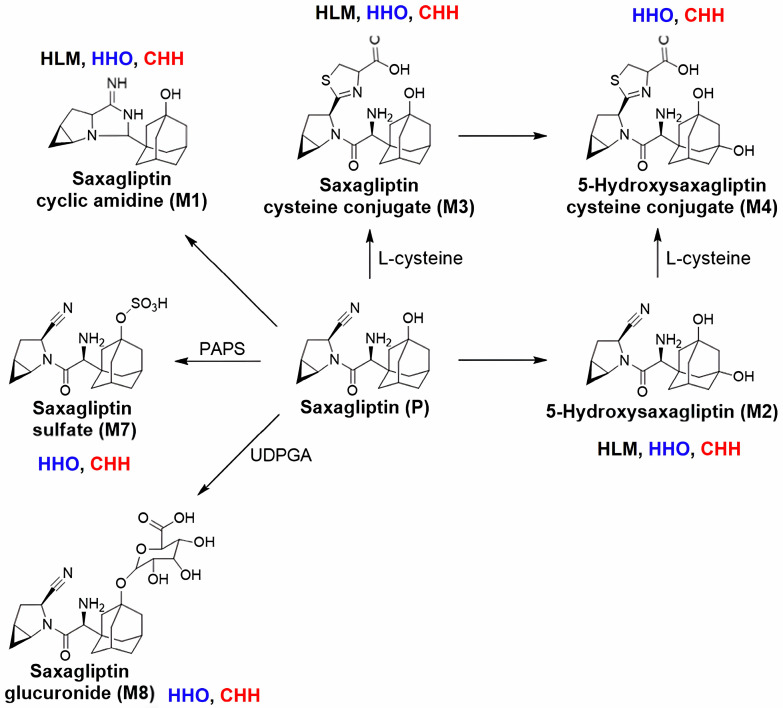
Metabolic pathways of saxagliptin in HLMs, HHOs, and CHHs. Abbreviations: HLMs, human liver microsomes; HHOs, human hepatic organoids; CHHs, cryopreserved human hepatocytes.

M4, M7, and M8 were identified in HHOs and CHHs, and the relative abundance of M1, M4, and M8 in HHOs was greater than that in CHHs, suggesting higher metabolic activity of nonenzymatic cyclic amidation, cysteine conjugation, and glucuronidation in HHOs. The higher abundance of the sulfate metabolite M7 suggested preferential sulfation in CHHs. The formation of M2, representing CYP3A4-mediated hydroxylation of saxagliptin [40], was comparable between HHOs and CHHs. Overall, the metabolite profiles of saxagliptin observed in CHHs were similar to those in HHOs, with nonenzymatic, phase I, and phase II metabolites being formed in both HHOs and CHHs compared to HLMs without adding specific cofactors (Figure 7).

## 4. Discussion

Identifying reactive metabolites during drug development is a critical safety step, as these short-lived electrophiles trigger DILI through protein adduct formation and oxidative stress [41]. Owing to their instability, reactive metabolites are identified via GSH or cysteine adducts. HLMs are widely used because they support early-stage metabolic mapping and enable GSH-conjugate identification with added NADPH and GSH [42,43]. Given the growing need for in vitro DILI assessment, plateable CHHs are considered the gold standard as they retain key metabolic enzymes (CYPs, UGTs, and SULTs) and transporters. Unlike suspension hepatocytes, plateable CHHs can be thawed and cultured in Matrigel-based 3D systems, allowing chronic dosing over several days [15,44,45].

We recently developed HHOs showing robust expression and activity of major CYPs, including CYP1A2, CYP2C9, CYP2C19, CYP2D6, and CYP3A4. CYP3A4 expression level in HHOs reaches ~30–40% of that observed in CHHs, whereas CYP2C19 and CYP2C9 show similar expression levels and metabolic activity in both systems [23]. Moreover, HHOs exhibit similar or higher sensitivity to toxicity than CHHs from known DILI-causing drugs (troglitazone, nefazodone, ketoconazole, and amitriptyline) with specific responses compared to negative structural analogs (rosiglitazone, buspirone, fluconazole, and chlorpromazine) [23].

Using these in vitro systems, we investigated reactive metabolites or their GSH or cysteine adducts of AAP, a well-known hepatotoxic agent, and saxagliptin, a potential cysteine-conjugate-forming drug, in HHOs, comparing the results with HLMs and CHHs. After incubating 50 μM AAP with HHOs for 24 h, NAPQI, AAP–GSH, AAP–G, and AAP–S were detected without exogenous cofactors (Figure 1). These findings highlight HHOs as a potential alternative in vitro system for metabolic mapping and GSH-conjugate identification. Moreover, HHOs exhibit concentration-dependent CYP1A2 induction after 48 h of omeprazole treatment, without affecting cell viability up to 100 μM. The EC_50_ value for CYP1A2 induction in HHOs is similar to that in CHHs, the gold standard for evaluating drug-metabolizing enzyme induction, although CYP1A2 activity in HHOs is ~2-fold lower than that in CHHs (Table 1; Figure 3). Accordingly, AAP and AAP–GSH formation increase with CYP1A2 upregulation in HHOs and CHHs. Collectively, this study showed that HHOs, including CHHs, can serve as in vitro screening systems to identify reactive metabolites in response to metabolic enzyme modulators such as the CYP1A2 inducer omeprazole, as both can be incubated with drugs or modulators for multiple days. However, phase II metabolites (AAP–G and AAP–S) differ between HHOs and CHHs. After 4 h incubation with 20 μM phenacetin, HHOs contained AAP–G and AAP–S, whereas CHHs contained only AAP–S (Figure 2C). Riches et al. [44] report that AAP sulfation is a high-affinity, low-capacity pathway. At low doses (<0.3 mM), AAP–S often predominates over AAP–G, with an intrinsic clearance three times higher than that of glucuronidation and substantial inter-individual variability (e.g., 0.13 ± 0.03 μL/min [range 0.04–0.29] for sulfation versus 0.06 ± 0.04 μL/min [range 0.02–0.16] for glucuronidation) [45]. At 20 μM phenacetin, AAP sulfation likely exceeds AAP glucuronidation, producing AAP–S without detectable AAP–G in CHHs. Higher SULT1A1 activity in CHHs supports the predominant formation of AAP-S in this system (Figure 2B). In contrast, basal UGT1A1 and UGT1A6 activities in HHOs were similar to or higher than those in CHHs (Figure 2B), consistent with greater AAP-G formation in HHOs (Figure 2C). Consequently, HHOs produced both phase II metabolites, confirming their suitability as an alternative in vitro system to mimic the in vivo environment.

Of the tested DPP-4 inhibitors (vildagliptin, saxagliptin, trelagliptin, linagliptin, and gemigliptin), vildagliptin and saxagliptin form cysteine or GSH conjugates [18,19,46]. Vildagliptin induces idiosyncratic hepatotoxicity, likely via an immune-mediated mechanism triggered by reactive metabolites [47]. Recently, Kim et al. report that saxagliptin forms reactive metabolites, including cysteine and GSH conjugates, in rat liver microsomes and in vivo, which may enhance DILI [18]. The key mechanism involves the cyano group (–CN) of vildagliptin and saxagliptin reacting with cysteine residues in endogenous proteins to form stable covalent adducts [47]. In our viability test, saxagliptin decreased cell viability in a concentration-dependent manner, with IC_50_ values of 117 μM and 144 μM in HHOs and CHHs, respectively. Furthermore, saxagliptin exhibited cytotoxicity in SW620, HCT116, SW480, and Caco2 colorectal cancer cells, with IC_50_ values of 168–500 μM [48].

To assess the potential contribution of saxagliptin reactive metabolites to hepatotoxicity, we investigated its metabolite profile in HLMs, HHOs, and CHHs. In CHHs and HHOs, saxagliptin was metabolized to M1, M2, M3, M4, M7, and M8 (Figure 6 and Figure 7). In contrast to in vivo rats, GSH conjugates of saxagliptin and M2 were absent in HHOs or CHHs, with only a trace amount of M3 (saxagliptin cysteine conjugate) detected. However, M4 (saxagliptin cysteine conjugate) formation was higher in HHOs than in CHHs. Mizuno et al. report that vildagliptin rapidly reacted with L-cysteine, while incubation with GSH did not significantly reduce its concentration, indicating a preference for cysteine over GSH [17]. Similar to the case of vildagliptin, saxagliptin favors cysteine over GSH in HHOs and CHHs. Additionally, covalent bonding with cysteine residues is thought to underlie rare immune-mediated hepatotoxicity caused by vildagliptin [17,46,47]. Although Su et al. did not detect cysteine or GSH conjugates of saxagliptin and 5-hydroxysaxagliptin in five normal participants during the phase 0 pharmacokinetic study [39], their ability to form reactive thiol adducts highlights the need to monitor liver-related adverse events at higher saxagliptin concentrations.

In conclusion, detecting NAPQI and AAP–GSH, along with elevated 5-hydroxysaxagliptin cysteine conjugates, suggests that HHOs can serve as an effective in vitro screening platform for reactive metabolites, similar to CHHs. However, comparisons between HLMs and cell-based systems require caution. Incubation time differences (1 h for HLMs, 24 h for HHOs/CHHs) and exogenous GSH in HLMs directly influence metabolite formation and trapping. Thus, comparisons between these systems should be interpreted qualitatively, not quantitatively.

## Figures and Tables

**Table 1:** Induction parameters for CYP1A2 after 48 h of omeprazole treatment in HHOs and CHHs.

System	Batches	Induction Parameters		
		EC_50_ (μM)	E_max_ (Fold-Increase)	Activity (E_max_/EC_50_)
HHOs	HDF HHO-P14	25.8	27.6	1.07
	HDF HHO-P38	7.57	17.1	2.26
	H9 HHO-P23	14.5	34.2	2.36
	Mean ± SD (*n* = 3)	16.0 ± 9.20	26.3 ± 8.63	1.90 ± 0.72
CHHs	Lot No. 303	13.8	50.9	3.70
	Lot No. 321	8.17	35.0	4.29
	Lot No. 337A	10.4	35.2	3.38
	Mean ± SD (*n* = 3)	10.8 ± 2.82	40.4 ± 9.15	3.79 ± 0.46 *

Data are presented as mean ± SD (*n* = 3). EC_50_ and E_max_ calculated from Figure 3C. * *p <* 0.05 compared with HHOs. Abbreviations: HHOs, human hepatic organoids; CHHs, cryopreserved human hepatocytes; CYP1A2, cytochrome P450 1A2; EC_50_, omeprazole concentration producing 50% of E_max_; E_max_, maximum fold induction of CYP1A2 mRNA.

## Data Availability

The original contributions presented in this study are included in the article/Supplementary Material. Further inquiries can be directed to the corresponding authors.

## Supplementary Materials

The following supporting information can be downloaded at https://www.mdpi.com/article/10.3390/pharmaceutics18040483/s1. Table S1. Primers used in RT-qPCR. Figure S1. Differentiation of hepatic organoids from HDF hPSCs and H9 hESCs. (A) Schematic of HHO differentiation. (B) RT-qPCR analysis of hepatic marker gene expression in HDF HHO (P11, P36, and P38), H9 HHO (P5, P12, and P13), and CHH 303. Data are presented as the mean ± SD (*n* = 3). (C) Representative immunofluorescence images of hepatic markers in HDF HHO-P15 and H9 HHO-P21. Figure S2. Extracted ion chromatograms of saxagliptin and metabolites in HHOs and CHHs after 1 h and 24 h, respectively (A) saxagliptin and M1 blank control, (B) M2 blank control, (C) M3 blank control, (D) M4 blank control, (E) M5, (F) M5 blank control, (G) M6, (H) M6 blank control, (I) M7 blank control, and (J) M8 blank control.
