# Supplementary material for: Identification of Reactive Metabolites of Acetaminophen and Saxagliptin in Human Hepatocytes and Hepatic Organoids
Source: Pharmaceutics. 2026 Apr 14;18(4):483. doi: 10.3390/pharmaceutics18040483 (PMC13119268; doi:10.3390/pharmaceutics18040483)
Supplement: Supplementary file 1 [file pharmaceutics-18-00483-s001.zip › pharmaceutics-4192980-supplementary.pdf]

pharmaceutics

Article

Identification of Reactive Metabolites of Acetaminophen and Saxagliptin in Human Hepatocytes and Hepatic Organoids

Im-Sook Song [1,2,†], Minyeong Pang [3,†], Min Seo Lee [4], Jihoon Lee [1,2], Kwang-Hyeon Liu [1], Min-Koo Choi [3], Han-Jin Park [5], Hyemin Kim [5,*] and Hye Suk Lee [4,*]

1 College of Pharmacy and Research Institute of Pharmaceutical Sciences, Kyungpook National University, Daegu 41566, Republic of Korea; isssong@knu.ac.kr (I.-S.S.); legadema0905@knu.ac.kr (J.L.); dstlkh@knu.ac.kr (K.-H.L.)
2 BK21 FOUR Community-Based Intelligent Novel Drug Discovery Education Unit, Vessel-Organ Interaction Research Center (VOICE), Kyungpook National University, Daegu 41566, Republic of Korea
3 College of Pharmacy, Dankook University, Cheon-an 31116, Republic of Korea; mignon@dankook.ac.kr (M.P.); minkoochoi@dankook.ac.kr (M.-K.C.)
4 Drug Metabolism and Bioanalysis Laboratory, College of Pharmacy, The Catholic University of Korea, Bucheon 14662, Republic of Korea; minseo.lee@catholic.ac.kr
5 Department of Predictive Toxicology, Korea Institute of Toxicology, Daejeon 34114, Republic of Korea; hjpark@kitox.re.kr
* Correspondence: sianalee@catholic.ac.kr (H.S.L.); hyeminkim@kitox.re.kr (H.K.); Tel.: +82-2-2164-4061 (H.S.L); +82-42-610-8312 (H.K.); Fax: +82-32-342-2013 (H.S.L); +82-42-610-8157 (H.K.)
† These authors contributed equally to this work.

Supplementary Table S1. Primers used in RT-qPCR

Gene	NCBI No.	Forward primer	Reverse primer	Product size (bp)
GAPDH	NM_002046	catgagaagtatgacaacagcct	agtccttccacgataccaaagt	113
ALB	NM_000477	gagaccagaggttgatgtgatg	agttccggggcataaaagtaag	114
AAT	NM_000295	gaagtcaaggacaccgagga	gctggcagaccttctgtctt	261
HNF4A	NM_000457	cgaaggtcaagctatgaggaca	atctgcgatgctggcaatct	141
TDO2	NM_005651	caaatcctctgggagttgga	gtccaaggctgtcatcgtct	165
MDR1	NM_000927.4	gggagcttaacacccgactta	gccaaaatcacaagggttagctt	154
MRP3	NM_001144070.1	caccaactcagtcaaacgtgc	gcaagaccatgaaagcgactc	197

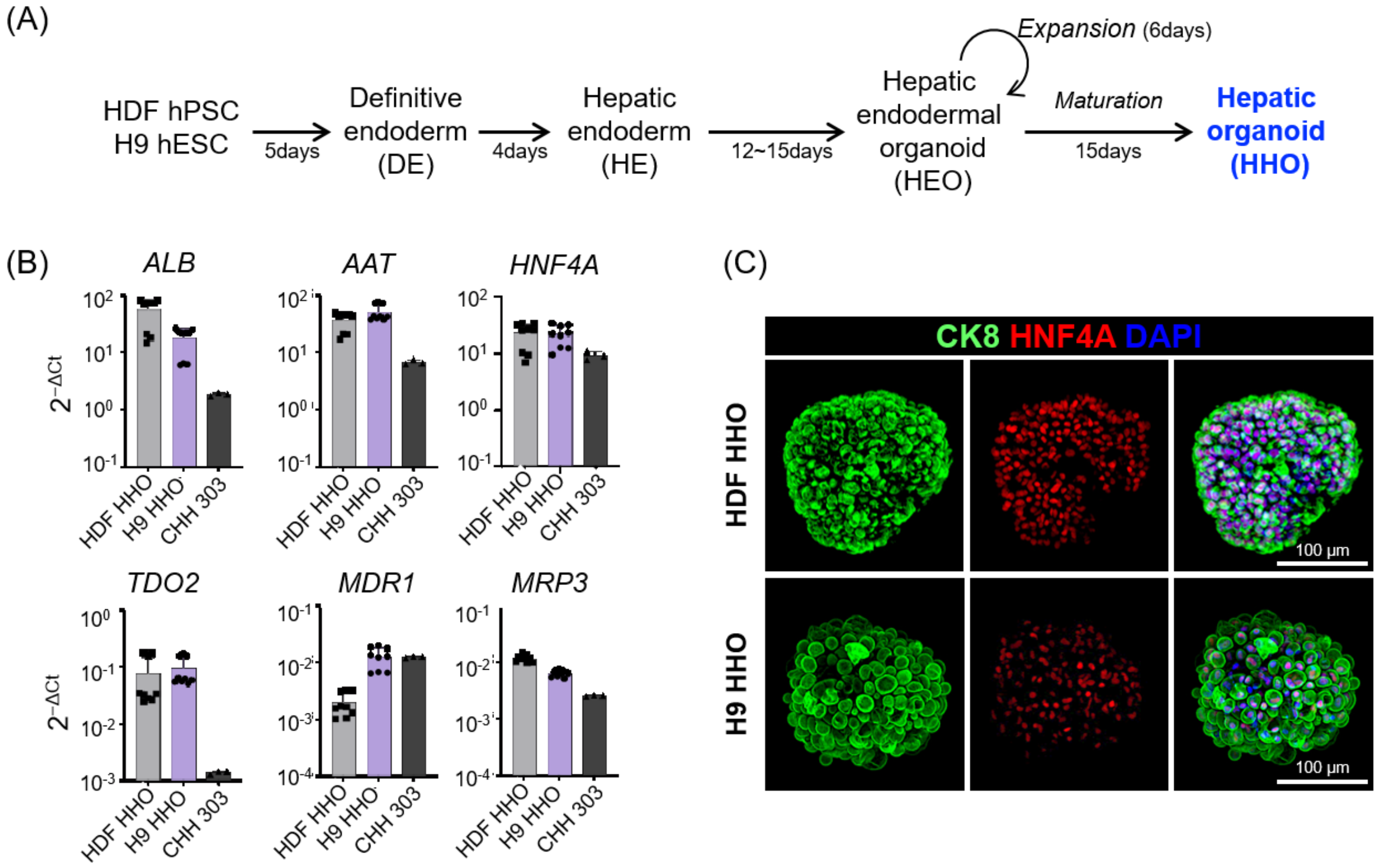

Supplementary Figure S1. Differentiation of hepatic organoids from HDF hPSCs and H9 hESCs. (A) Schematic of HHO differentiation. (B) RT-qPCR analysis of hepatic marker gene expression in HDF HHO (P11, P36, and P38), H9 HHO (P5, P12, and P13), and CHH 303. Data are presented as the mean ± SD (n = 3). (C) Representative immunofluorescence images of hepatic markers in HDF HHO-P15 and H9 HHO-P21. Abbreviations: hPSC, human induced pluripotent stem cells; hESC, human embryonic stem cells; HHOs, human hepatic organoids; CHHs, cryopreserved human hepatocytes.

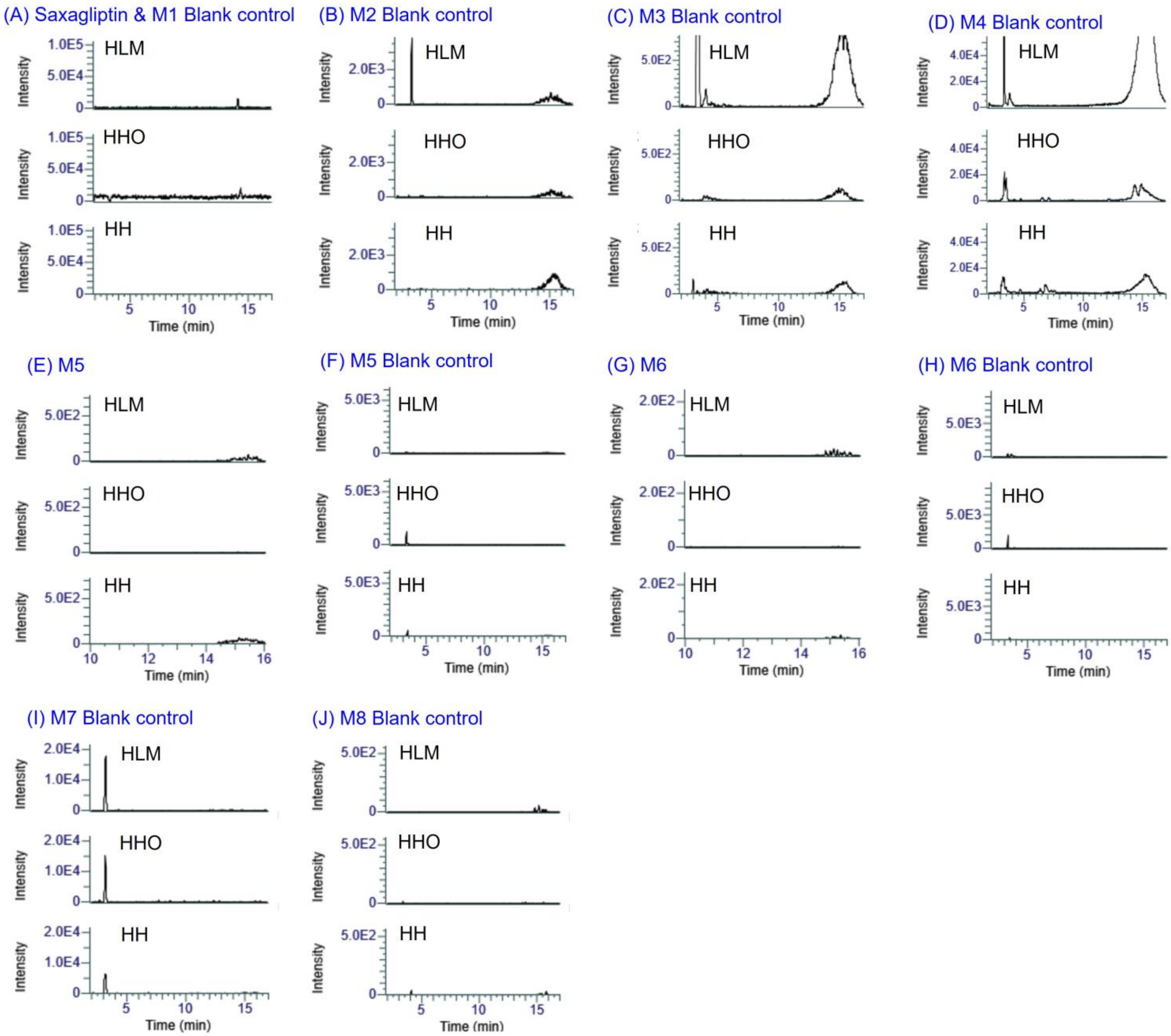

Supplementary Figure S2. Extracted ion chromatograms of saxagliptin and metabolites in HHOs and CHHs after 1 h and 24 h, respectively (A) saxagliptin and M1 blank control, (B) M2 blank control, (C) M3 blank control, (D) M4 blank control, (E) M5, (F) M5 blank control, (G) M6, (H) M6 blank control, (I) M7 blank control, and (J) M8 blank control. Abbreviations: HLMs, human liver microsomes; HHOs, human hepatic organoids; CHHs, cryopreserved human hepatocytes.
